# The complete chloroplast genome of *Castanopsis mekongensis* A. Camus (Fagaceae)

**DOI:** 10.1080/23802359.2020.1860707

**Published:** 2021-01-16

**Authors:** Jingyu Peng, Lin Yang, Anan Duan, Dawei Wang, Siguang Li

**Affiliations:** a Key Laboratory for Forest Resources Conservation and Utilization in the Southwest Mountains of China Ministry of Education, Southwest Forestry University, Kunming, China; b Yunnan Academy of Forestry and Grassland, Kunming, China

**Keywords:** *Castanopsis mekongensis* A. Camus, chloroplast genome, phylogenetic analysis

## Abstract

*Castanopsis mekongensis* is highly valued in the furniture industry for its good wood texture, an endemic species in Yunnan province, southwest China. In our study, the chloroplast genome of *C. mekongensis* was assembled and characterized based on Illumina pair-end sequencing data. The total genome size of *C. mekongensis* was 160,699 bp, displayed a typical quadripartite structure including a pair of inverted repeat (IRs, 25,714 bp) regions separated by a large single-copy (LSC, 90,278 bp) region and a small single-copy (SSC, 18,963 bp) region. The overall guanine–cytosine (GC) content was 36.8%. We annotated 130 genes in the genome, containing 85 protein-coding genes, 37 tRNA genes, and eight rRNA genes, 12 genes contain a single intron, and two genes have two introns. The result of phylogenetic analysis based on maximum-likelihood (ML) phylogenetic tree indicated that *C. mekongensis* was most closely related to *C. hainanensis*.

*Castanopsis mekongensis* is an evergreen plant that belongs to the family Fagaceae, which is mainly distributed in Yunnan province, southwest China (Xia and Tong 1998). *Castanopsis mekongensis* is highly valued in the furniture industry for its good wood texture (Duanmu 1996). However, the yield of wild *C. mekongensis* gradually decreases due to the slow growth rate and excessive excavation (Duanmu 1996). To restore its genetic diversity and to solve the market demand, it is necessary to protect the remaining wild resources. As such, to better develop effective protecting strategies, we should understand the phylogenetic relationships between *C. mekongensis* and other *Castanopsis* species in the first step.

The chloroplast owning one circular DNA molecule is a semi-autonomous cell organelle in higher plants, which play a significant role in photosynthesis (Isao et al. 2010). Due to its stable structure and uniparental inheritance, the chloroplast genome is extremely useful for studying the origin and evolution of plants at different taxonomic levels (Osuna-Mascaró et al. 2018). In recent years, the chloroplast genome of many species was used to demonstrate considerable phylogenetic information (Xue et al. 2019; Zhang et al. 2019), while the chloroplast genomes of *C. mekongensis* is still unknown. In this paper, we reported the complete chloroplast genome sequence of *C. mekongensis* employing high-throughput sequencing. Phylogenetic analysis was conducted to explore its phylogenetic relationships with other relatives.

The leave samples of *C. mekongensis* were collected from Kunming, Yunnan, China (102°44′38″ E, 25°8′6″ N, 1928 m). Total DNA was extracted using a Wolact Magnetic beats Plant DNA Purification Kit (KLink Biotechnologies, Shenzhen, China), the voucher specimens of *C. mekongensis* were deposited at the herbarium of Southwest Forestry University, Kunming, China (accession number: SWFU-FAG-FCM-5825). Genomic DNA was sequenced using the Illumina Novaseq 6000 platform at Annoroad Gene Technology (Beijing, China). About 5.0 GB high-quality clean reads were generated with adaptors trimmed. Then, the complete chloroplast genome was assembled by Get Organelle v1.6.2 (Jin et al. 2020). Genome annotation was performed by Geneious R8 (Kearse et al. 2012) and manually adjusted by comparing it to the reference chloroplast genome of *C. hainanensis* (GenBank accession number MG383644.1). The annotated genomic sequence has been submitted to GenBank (accession number: MW043480).

The complete chloroplast genomes of *C. mekongensis* displayed the typical quadripartite structure with the length of 160,699 bp, including a large single copy (LSC) region of 90,278 bp, a small single copy (SSC) region of 18,963 bp, and a pair of inverted repeats (IRs) region of 25,714 bp. The guanine–cytosine (GC) content of chloroplast genomes is 36.8%; the GC content of the IR regions is 42.8% and those of LSC and SSC regions 34.6% and 30.9%, respectively. The chloroplast genes of *C. mekongensis* contain 130 functional genes, including 85 protein-coding genes, eight rRNA genes, and 37 tRNA genes. There are 12 genes (atpF, ndhB, petB, rpl2, rpoC1, rps16, trnA-UGC, trnG-GCC, trnI-GAU, trnK-UUU, trnL-UAA, and trnV-UAC) contain a single intron, and two genes (ycf3 and clpP) have two introns.

The chloroplast genomes of *C. mekongensis* in this study and 23 previously reported species of Fagales were aligned by MAFFT v7 using default settings (Katoh and Standley 2013). The maximum-likelihood (ML) phylogenetic tree based on complete chloroplast genome sequences was performed by IQ-tree 1.5.5 (Nguyen et al. 2015). The optimal nucleotide substitution model (TVM + F+R7) was selected by ModelFinder (Kalyaanamoorthy et al. 2017). The ML analyses were conducted with 1000 rapid bootstrap replicates along with a search for the best-scoring ML tree (Gao et al. 2018). The phylogenetic analysis showed that *C. mekongensis* appeared to be most closely related to *C. hainanensis* (Figure 1). The chloroplast genome of *C. mekongensis* will provide useful genetic information for understanding the evolutionary relationships and conservation management.

**Figure 1.**
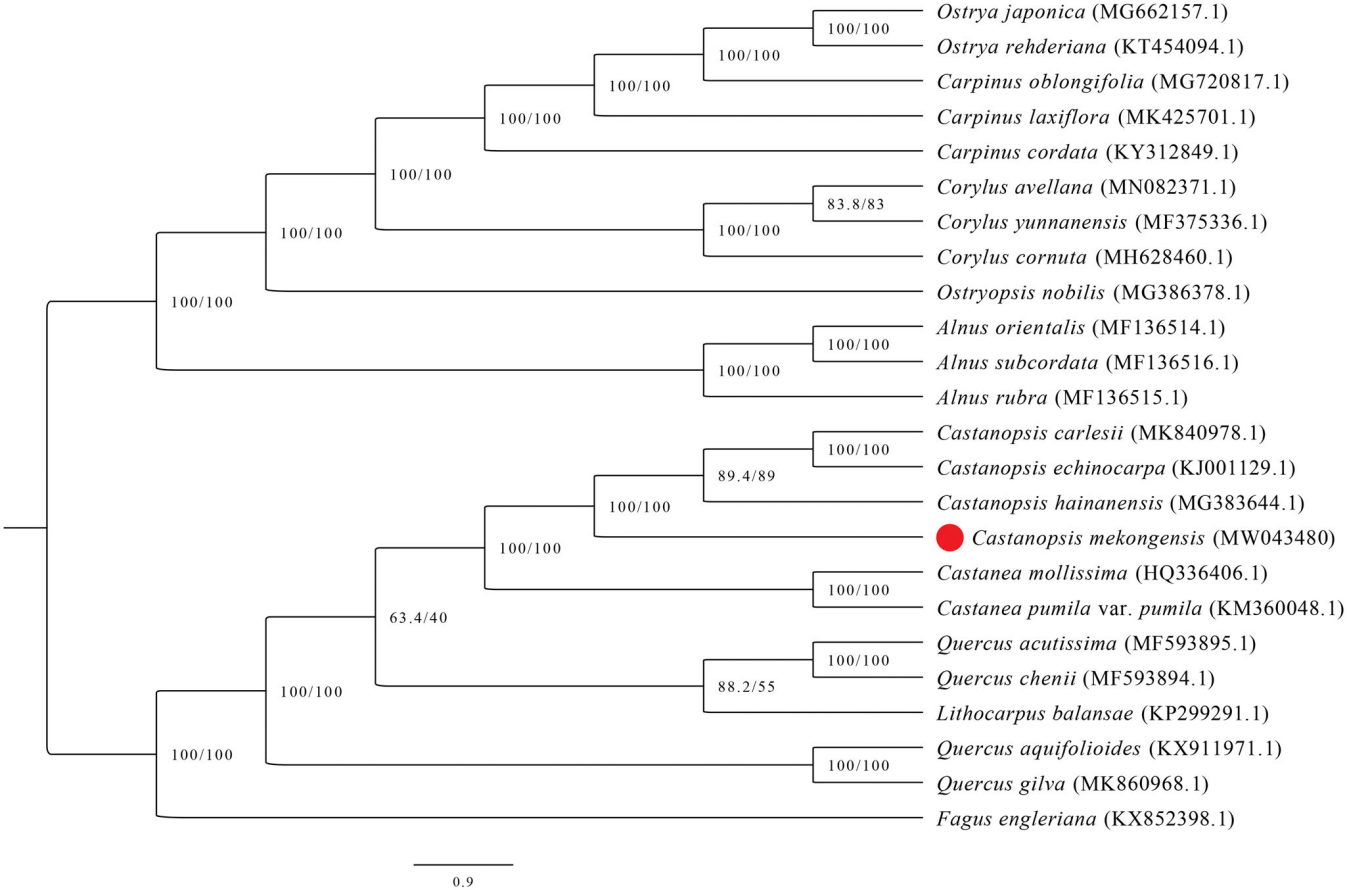
Phylogenetic relationships among 24 complete chloroplast genomes (*Castanopsis mekongensis* in this study and 23 previously reported species) of Fagales. Bootstrap values based on 1000 replicates were provided near branches.

## Data Availability

The voucher specimens of *Castanopsis mekongensis* were deposited at the herbarium of Southwest Forestry University, Kunming, Yunnan, China (accession number: SWFU-FAG-FCM-5825). The complete chloroplast genome sequence of *C. mekongensis* in this study has been submitted to National Center for Biotechnology Information (NCBI) (https://www.ncbi.nlm.nih.gov/) and obtained the GenBank accession number (MW043480).
